# Distinct reduction in relative microglial glucose uptake compared to astrocytes and neurons upon isolation from the brain environment

**DOI:** 10.3389/fncel.2025.1572431

**Published:** 2025-09-15

**Authors:** Sebastian T. Kunte, Johannes Gnörich, Philipp Beumers, Laura M. Bartos, Stephan Wagner, Karin Wind-Mark, Adrien Holzgreve, Dennis Pötter, Rudolf A. Werner, Sibylle Ziegler, Nathalie L. Albert, Alessio Colombo, Sabina Tahirovic, Matthias Brendel

**Affiliations:** 1 Department of Nuclear Medicine, LMU University Hospital, Munich, Germany; 2 Section of Clinical and Comparative Neuropathology, Institute for Veterinary Pathology, Center for Clinical Veterinary Medicine, LMU, Munich, Germany; 3 Munich Cluster for Systems Neurology (SyNergy), University of Munich, Munich, Germany; 4 Russell H. Morgan Department of Radiology and Radiological Sciences, Johns Hopkins School of Medicine, Baltimore, MD, United States; 5 German Cancer Consortium (DKTK), Partner Site Munich, German Cancer Research Center (DKFZ), Heidelberg, Germany; 6 Bavarian Cancer Research Center (BZKF), Erlangen, Germany; 7 German Center for Neurodegenerative Diseases (DZNE) Munich, Munich, Germany

**Keywords:** microglia, glucose uptake, *in vivo*, *in vitro*, scRadiotracing

## Abstract

**Introduction:**

Microglial energy metabolism has gained attention for the treatment of neurodegenerative diseases. *In vitro* methods provide important insights; however, it remains unclear whether the metabolism of highly motile microglia is preserved outside their regular environment. Therefore, we directly compared the microglial glucose uptake *in vivo* and in vitro in mice.

**Methods:**

Microglia and astrocytes were isolated from the brain using immunomagnetic cell sorting following [^18^F]FDG injection in living mice, followed by gamma and single-cell radiotracing (scRadiotracing). Enriched cell fractions were incubated with excess [^18^F]FDG (50,000-fold) *in vivo*, washed, and measured equivalently. For all fractions, radioactivity per cell was normalized to the injected or incubated radioactivity, and ratios of microglialuptake were calculated relative to astrocytes and the microglia/astrocyte-negative fraction. The experiment was repeated using a glucose-free buffer and validated by in vitro incubation without prior in vivo [^18^F]FDG injection to exclude the influence of fasting and glucose injection.

**Results:**

scRadiotracing results were compared against cell culture [^18^F]-FDG incubation. The in vivo glucose uptake of microglia was higher when compared to astrocytes (50.4-fold, *p* < 0.0001) and non-microglia/ non-astrocyte cells (10.6-fold, *p* < 0.0001). Microglia still exhibited the highest glucose uptake in vitro, but with a distinct reduction in microglia-to-astrocyte (5.7-fold, *p* < 0.0015) and microglia-to-microglia/astrocyte-negative ratios (1.7 fold, *p* < 0.0001). Fasting and in vitro incubation were used to validate the results. Cell culture indicated low microglial uptake compared to that in neurons (1:100) or astrocytes (1:10).

**Discussion:**

Compared to astrocytes and other cells, microglia show a distinct reduction in uptake in vitro compared to in vivo uptake. Our results emphasize that in vitro experiments should be interpreted with caution when studying microglial energy metabolism.

## 1 Introduction

Microglial activation and characteristic metabolic patterns have been widely recognized as hallmarks of neurodegenerative diseases such as Alzheimer’s disease (AD) (Gao et al., 2019; Rauchmann et al., 2022). These alterations may substantially precede the manifestation of pathognomonic cerebral alterations, i.e., the presence of neurofibrillary tangles or senile plaques (Bubber et al., 2005; Mattson, 2004; Moreira et al., 2007; Wallace, 2005). Therefore, fundamental knowledge of changes in cellular metabolism is crucial to facilitate the recognition of disease onset at an early stage (Mosconi, 2005). Additionally, future investigations on pivotal microglial functions and metabolic interactions between resident cells of the brain may yield novel therapeutic approaches (Fumagalli et al., 2018; Jung et al., 2025). In this regard, the assessment of *in vivo* microglial metabolism remains challenging, thereby hindering the progress in understanding the observed metabolism-associated biomechanisms. The majority of studies conducted hitherto, thus, have focused on cultured cells and Seahorse assay-based bioenergetic analysis. The Seahorse assay facilitates the quantification of the oxygen consumption rate (OCR) as a measure of mitochondrial respiration and the extracellular acidification rate (ECAR), indicating glycolysis (Zhang et al., 2021). In addition, single-cell RNA sequencing (scRNA-seq) analysis captures changes in key metabolic enzymes that influence changes in cellular energy metabolism (Hrovatin et al., 2022). However, microglia are capable of rapidly shifting between activation states in response to their environment. Consequently, they need to specialize their metabolic demands to suit these distinct states, such as resting, surveillance, or activation (Bernier et al., 2020; Orihuela et al., 2016). Several studies uncovered distinct expression patterns of key metabolic enzymes and transporters in microglia, dependent on cellular activation, by scRNA-seq and protein measurements (Xiang et al., 2021; Hammond et al., 2019; Hrovatin et al., 2022; Olah et al., 2020; Zhang et al., 2014).

However, microglial energy metabolism assays that use a snapshot several hours after isolation of microglia from the regular brain environment may still experience bias due to changes in cellular energy demand and gene expression. Thus, although these assays are highly valuable for comparison of microglial energy metabolism under different conditions, there is a strong need to investigate alterations in microglial energy metabolism *in vivo* and *in vitro*. Radioactive 2-Fluor-2-desoxy-D-glucose (i.e., [^18^F]FDG) facilitates the assessment of cellular glucose uptake at the time of tracer injection *in vivo* because FDG is trapped inside the cell upon phosphorylation and is not further metabolized. Importantly, this methodology can also be used in conjunction with PET (Minoshima et al., 2022) or autoradiography (Sokoloff et al., 1977) to generate a snapshot of glucose uptake during a certain task. Moreover, we and others recently established workflows to track this tracer uptake at cellular resolution (Bartos et al., 2022; Choi et al., 2021; Xiang et al., 2021). In particular, two independent studies observed a significant increase in microglial glucose uptake in mouse models of amyloidosis when compared to wild-type mice, which indicated an altered energy demand at different microglial activation states (Xiang et al., 2021; Choi et al., 2021).

To compare glucose uptake by microglia *in vivo* and *in vitro*, we extended our established workflow and performed a head-to-head comparison of tracer uptake per cell between *in vivo* [^18^F]FDG injection in living mice and subsequent *in vitro* incubation of the same *ex vivo* isolated cells with excessive [^18^F]FDG. Furthermore, we performed cross-validation of our results using a glucose-free buffer and *in vitro* application without prior *in vivo* [^18^F]FDG injection. Finally, we compared microglial glucose uptake in cell cultures with that by microglia in a regular brain environment.

## 2 Materials and methods

### 2.1 Experimental setup and study design

**Figure 1:**
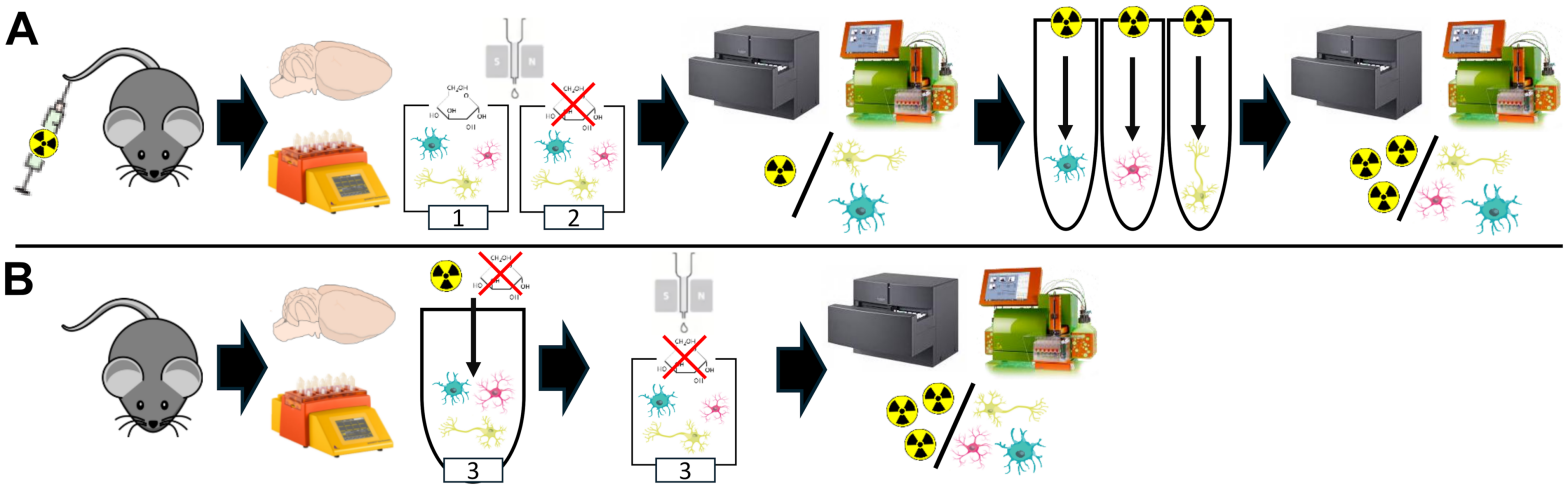
Experimental setup for head-to-head comparison of *in vivo* and *in vitro* glucose uptake using a modified scRadiotracing protocol. **(A)** The experimental workflow of an extended scRadiotracing protocol enables direct comparison of *in vivo* and *in vitro* glucose uptake (study group 1, Table 1). *In vivo* [^18^F]FDG application was followed by brain dissociation in a buffer containing glucose, magnetic-activated cell sorting, and determination of radioactivity per cell body. Radioactivity per cell body was equivalently determined after subsequent *in vitro* incubation with excessive [^18^F]FDG. To investigate the influence of strict fasting on cells, a glucose-free buffer was used equally throughout the workflow (study group 2, Table 1). **(B)** To exclude potential effects of *in vivo* [^18^F]FDG application prior to *in vitro* incubation, the scRadiotracing workflow was modified for *in vitro* incubation without prior *in vivo* [^18^F]FDG injection (study group 3, Table 1). After brain extraction and dissociation, *in vitro* incubation was performed, followed by magnetic-activated cell sorting and determination of the radioactivity per cell body. Glucose-free buffer was used throughout the workflow.

All animal experiments were performed with the approval of the local animal care committee of the Government of Upper Bavaria (Regierung von Oberbayern) and in accordance with the National Guidelines for the Protection of Animals. The animals used for the experiments were monitored by a veterinarian. Experiments were executed in compliance with the ARRIVE (Animal Research: Reporting of *In Vivo* Experiments) guidelines and in accordance with the U. K. Animals (Scientific Procedures) Act, 1986, and associated guidelines, EU Directive 2010/63/EU for animal experiments. Animals were kept in a temperature- and humidity-controlled environment with a 12-h light–dark cycle, with unrestricted access to food and water. For this study, a total of female (*n* = 20) wild-type mice (C57BL6) at an average age of 3.4 ± 0.4 months underwent single-cell Radiotracing (scRadiotracing) (Bartos et al., 2022; Xiang et al., 2021) for direct comparison of *in vivo* and *in vitro* glucose uptake of microglia. Figure 1 illustrates the study design, study groups, and workflow, which included tracer injection, brain removal, generation of single-cell suspension, immunomagnetic cell sorting (MACS), gamma emission counting, and flow cytometry. Sample sizes were determined based on previous experience with the workflow (Bartos et al., 2024a; Dinkel et al., 2024; Gnörich et al., 2023; Xiang et al., 2021; Slemann et al., 2024; Bartos et al., 2023; Bartos et al., 2024b).

Tracer uptake upon *in vivo* [^18^F]FDG injection, followed by incubation with excess [^18^F]FDG (50,000-fold), was measured in mice (*n* = 16). Using catheters, the tracer was injected into the ventral tail vein, followed by flushing to ensure complete bolus application (Table 1). The mice were separated from their access to food for >3 h before tracer injection. Anesthesia was performed with 1.5% isoflurane delivered at 1.5 L/min O_2_. To consider the influence of cell fasting, a buffer containing glucose (1,000 mg/L) and a glucose-free buffer were used for each mouse (n = 8).

**Table 1:** Summary of study groups.

Method	Group	Buffer	Mouse model	Animals (n)	Sex	Age (months)	Body weight (g)	Injected dose (MBq)	Incubation dose (MBq)
*In vivo* + *In vitro*	1	glucose	wild-type	8	f	4.0	23.3 ± 1.0	15.0 ± 3.3	8.5 ± 0.5
	2	glucose-free	wild-type	8	f	3.5	21.3 ± 1.3	16.2 ± 1.6	2.9 ± 0.5
*In vitro* validation	3	glucose-free	wild-type	4	f	3.0	20.5 ± 0.6	/	7.3 ± 0.4
Cell culture	4	glucose-free	/	11–12 per cell type	f	/	/	/	0.1

Brief overview of key determinants defining the major study groups in the experimental setup of this study. f = female, MBq = megabecquerel.

For cross-validation, another mouse (n = 4) underwent *in vitro* [^18^F]FDG incubation without prior *in vivo* [^18^F]FDG injection after the generation of the single-cell suspension (glucose-free buffer).

Moreover, we measured the glucose uptake of cultured microglia, astrocytes, and neurons after [^18^F]FDG incubation for comparison with uptake values from scRadiotracing (Bartos et al., 2022).

### 2.2 Preparation of single-cell suspensions

Using the Adult Brain Dissociation Kit, mouse, and rat (Miltenyi Biotec, 130–107-677), single-cell suspensions were prepared according to the manufacturer’s instructions.

After dissection, adult mouse brains were briefly washed in cold phosphate-buffered saline (PBS) and cut into sagittal slices (*n* = 8). Brain dissociation was performed in enzyme mixes 1 and 2 using a gentleMACS™ Octo Dissociator with Heaters (Miltenyi Biotec, 130–096-427). The resulting brain homogenates were then applied onto pre-moistened MACS® SmartStrainers (70 μm) and washed by rinsing with 2 × 10 mL of PBS. After centrifugation at 300 × g for 10 min at 4 °C, the pellets were resuspended in PBS and Debris Removal Solution. Cold PBS was gently overlaid. After centrifugation at 3,000 × *g* for 10 min at 4 °C, the top two phases were discarded. The cell pellets were washed and resuspended in 1 mL of Red Blood Cell Removal Solution (10x). After incubation for 10 min in the dark in a refrigerator at 4 °C, cell pellets were washed with cold PBS-0.5% BSA buffer and collected for positive isolation of microglia and astrocytes via magnetic-activated cell sorting (Bartos et al., 2022; Xiang et al., 2021).

To consider the influence of fasting, this preparation process was performed with PBS buffer containing glucose for wild-type mice (*n* = 8) and with glucose-free buffer for wild-type mice (*n* = 8 + 4).

### 2.3 Isolation of microglia

The isolation process of microglia was performed by immunomagnetic cell sorting following the manufacturer’s instructions for CD11b (microglia) MicroBeads (Miltenyi Biotec, 130–093-634) for up to 10^7^ cells.

Prepared single-cell pellets were resuspended in 90 μL of cold PBS-0.5% BSA buffer and labeled with 10 μL of CD11b (Microglia) MicroBeads (Miltenyi Biotec, 130–093-634). After incubation for 15 min in the dark in a refrigerator at 4 °C, the cells were washed and resuspended in 500 μL of PBS-0.5% BSA buffer. LS columns (Miltenyi Biotec, 130–042-401) were prepared by rinsing with 3 mL of PBS-0.5% BSA buffer, followed by application of the cell suspensions onto the columns. The columns were washed with 3 × 3 mL of PBS-0.5% BSA buffer. The flow-through containing unlabeled cells was collected as the microglia-depleted fraction. Labeled cells were collected by flushing the columns with 5 mL of PBS-0.5% BSA buffer and were considered microglia-enriched fractions. Microglia-enriched fractions were analyzed using gamma emission recording (radioactivity per pellet, see Section 2.6) and flow cytometry (cell count and purity per pellet, see Section 2.7). Glucose-enriched or glucose-free buffers were maintained as described in Section 2.2.

### 2.4 Isolation of astrocytes

To isolate astrocytes by immunomagnetic cell sorting, the Anti-ACSA2 MicroBead Kit (Miltenyi Biotec, 130–097-678) was used according to the manufacturer‘s instructions for up to 10^7^ total cells.

The flow-through fractions containing unlabeled cells negative for CD11b from the microglial isolation process were resuspended in 80 μL of cold AstroMACS Separation Buffer (Miltenyi Biotec, 130–117-336) and labeled with 10 μL of FcR Blocking Reagent. After incubation for 10 min in the dark in a refrigerator, 10 μL of Anti-ACSA2 MicroBeads was added, followed by incubation for 15 min in a refrigerator. The cells were washed and applied to pre-moistened LS columns. The columns were washed with 3 × 3 mL of AstroMACS Separation Buffer. The flow-through containing unlabeled cells was collected as microglia- and astrocyte-depleted fractions. The predominant proportion of neuronal cells in this fraction (79.5 ± 5.0%) was validated by CD90.2 staining (see Section 2.7). Labeled cells were collected by flushing the columns with 5 mL of AstroMACS Separation Buffer and were considered as astrocyte-enriched fractions. Microglia- and astrocyte-depleted fractions, as well as astrocyte-enriched fractions, were analyzed using gamma emission recording (radioactivity per pellet, see Section 2.6) and flow cytometry (cell count and purity per pellet, see Section 2.7). Glucose-enriched or glucose-free buffers were maintained as described in Section 2.2. Validation of ACSA-2 sorting of astrocytes was validated via GFAP elsewhere (Xiang et al., 2021).

### 2.5 *In vitro* incubation

For a direct comparison of *in vivo* and *in vitro* glucose uptake, all *ex vivo* isolated cell fractions were resuspended in 1 mL PBS and incubated *in vitro* with excess [^18^F]FDG (50,000-fold; radioactivity used for incubation relative to radioactivity in the cell pellet). After 30 min of uptake in the dark, the samples were washed to remove non-cellular-bound tracers. Then, 5 mL of cold PBS was added, followed by centrifugation at 400 × g for 5 min at 4 °C. This washing procedure was repeated five times to ensure the exclusion of non-cellular [^18^F]FDG from the measured probes.

Glucose-enriched and glucose-free buffers were maintained as described in Section 2.2. To eliminate the confounding effects of the *in vivo* injection, the procedure was repeated by *in vitro* incubation without prior *in vivo* [^18^F]FDG injection in wild-type mice (*n* = 4). *In vitro* incubation was performed directly after the preparation of single-cell suspensions. Non-cellular bound [^18^F]FDG was removed during >5 washing steps during the isolation process for microglia and astrocytes.

### 2.6 Gamma emission measurements

To measure the radioactivity concentrations of the cell pellets, gamma emission recordings were performed using a highly sensitive gamma counter (Hidex AMG Automatic Gamma Counter, Mainz, Germany). Each probe was measured for 1 min. The results of cell sorting after the *in vivo* injection were normalized to the activity in the whole brain, including decay correction to the time of tracer injection. Results of cell sorting after *in vitro* incubation were normalized to the amount of radioactivity used for incubation.

### 2.7 Sample analysis by flow cytometry

Flow cytometry was performed to analyze the fraction purity and cell count after *in vivo* tracer injection and *in vitro* incubation.

**Figure 2:**
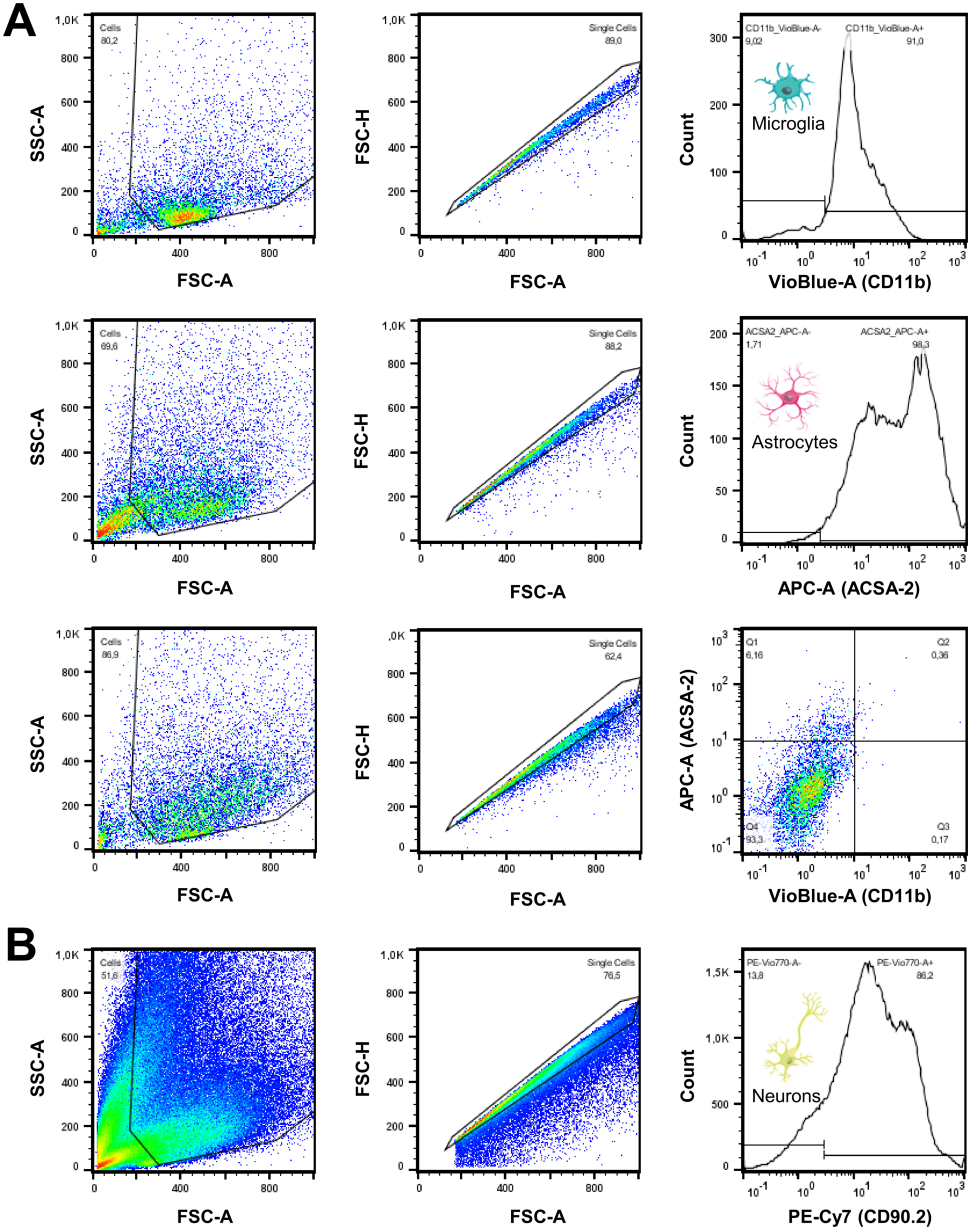
Quality measures of cell sorting procedures show excellent purity of enriched cell fractions. **(A)** Following the gating strategy, validation of purity and cell counting of isolated cell fractions (CD11b+, ACSA2+, CD11b-/ACSA2-) was executed by FACS analysis. As shown in the stagger-offset histograms, microglial and astrocytic fractions were collected with robust purity. Non-microglia/non-astrocyte cell fraction gates were defined as CD11b and ACSA2 negative. **(B)** The high proportion of neurons in non-microglia/non-astrocyte cell fractions was cross-validated using CD90.2 in independent samples. A high proportion of neurons was identified after manual microglia and astrocyte depletion and after depletion using an AutoMACS® Pro Separator.

The cell pellets were resuspended in 100 μL cold PBS. Microglia were stained with 1.5 μL of CD11b Antibody, linked with VioBlue® (Miltenyi Biotec, 130–113-810) or APC (BioLegend®, 101,212). Astrocytes were stained with 1.5 μL of ACSA2 Antibody, linked with APC (Miltenyi Biotec, 130–116-142) or PE-Vio® 615 (Miltenyi Biotec, 130–116-249). After incubation for 10 min in the dark in a refrigerator, the incubation was terminated by adding 1.5 mL of PBS. The samples were analyzed by flow cytometry using a MACSQuant® Analyzer 10 Flow Cytometer (Miltenyi Biotec, 130–096-343; Figure 2A). Cell count represents the number of singlets per fraction. Purity was defined as the percentage of CD11b-positive microglia or ACSA2-positive astrocytes per enriched cell fraction. Purity of the microglia- and astrocyte-depleted fraction was determined by CD11b- and ACSA2-negativity, and the proportion of neurons was cross-validated by CD90.2 (Thy-1.2 Antibody, Invitrogen, 25–0902-81; Figure 2B) staining using independent samples that underwent equal processing. CD11b-VioBlue® and APC-ACSA2 are presented throughout the manuscript.

### 2.8 Cell culture

Relative [^18^F]FDG uptake by cells in an exclusive *in vitro* environment was measured to compare the cell culture results with the uptake of cells isolated from a regular brain environment (see Sections 2.2–2.6, 2.7) performed in buffer without glucose. Primary embryonic hippocampal neurons and astrocytes were isolated from embryonic day 18 CD rats (Charles River), as previously described (Kaech and Banker, 2006; Montagna et al., 2019; Willem et al., 2015), and cultured in a humidified 5% CO_2_ incubator at 37 °C. Hippocampal neurons were plated onto 6-well plates containing glass coverslips coated with 1 mg/mL poly-L-lysine and maintained for 10 days in Neurobasal medium supplemented with 2% B27 and 0.5 mM L-glutamine (all from Invitrogen). Astrocytes were plated in untreated T-75 tissue culture flasks (Nunc) and cultured in minimal essential medium (Gibco) supplemented with horse serum (MEM-HS) until they reached approximately 80% confluency, when the cells were passaged and used 2 days after the first or second passaging. Postnatal P7 mouse microglia were isolated and cultured as previously described (Dinkel et al., 2024).

Wells were prepared with either microglia or astrocytes (*n* = 75,000 and 600 μL DMEM-F12 (Gibco) supplemented with 10% FBS and 1% penicillin/streptomycin (Gibco) medium per well) or neurons (*n* = 75,000 and 600 μL NEUROBASAL medium per well) and incubated for 60 min at 37 °C. After incubation for 120 min with [^18^F]FDG (100 kBq in 200 μL per well), the medium and tracers were removed. To eliminate non-cellular-bound tracers, the wells were washed with 800 μL of PBS. The cells were then incubated twice with 800 μL NaOH for 10 min and transferred to collection tubes. Cellular radioactivity was measured by gamma counting (see Section 2.6). Radioactivity values (counts per minute; CPM) were normalized to the measured reference activity (CPM/CPMref), including decay correction to the time of tracer incubation as a readout of the percentage uptake. Furthermore, cellular radioactivity was normalized to the cell number as uptake per cell (CPM/cell).

### 2.9 Statistical analysis

IBM® SPSS® Statistics (IBM, V28.0) and GraphPad Prism (V9.4) were used for the statistical analysis and visualization. A *p*-value of < 0.05 was considered significant.

For all cell fractions, radioactivity per cell was calculated and normalized to injected or incubated radioactivity, and ratios of glucose uptake in microglia cells were calculated relative to the uptake in astrocytes and the microglia/astrocyte-negative fraction.

Kolmogorov–Smirnov and Shapiro–Wilk tests were used to confirm the data of each group (see Section 2.1; Figure 1) on normal distribution. A paired Student’s t-test was used to compare the glucose uptake of microglia-to-astrocyte, microglia-to-microglia/astrocyte-negative, and astrocyte-to-microglia/astrocyte-negative cell ratios between *in vivo* injection and *in vitro* incubation (see 3.1). Unpaired Student’s t-tests were used to compare the absolute glucose uptake values between single microglia, single astrocytes, and non-microglia/non-astrocyte cells (see Section 3.2). A one-way ANOVA was used to compare glucose uptake by microglia, astrocytes, and neurons in Section 3.3. All data are presented as mean ± SD. A *post hoc* power analysis (two-tailed *t*-test, *α* = 0.05, power = 80%) indicated that the study was sufficiently powered to detect the actual effect sizes (all Cohen’s *d* > 1.16) for the comparison of *in vivo* and *in vitro* FDG uptake as well as the influence of fasting (with *n* = 8 samples per group). For the cell culture experiment, the required effect size of Cohen’s *d* > 0.55 (with *n* = 11–12 samples per group) to detect significant differences at a power of 80% and *α* = 0.05 (one-way ANOVA) was met.

## 3 Results

### 3.1 Distinct relative reduction of microglial glucose uptake when isolated from the brain environment

First, we aimed to compare the glucose uptake of microglia in the regular brain environment after *in vivo* tracer injection head-to-head with the glucose uptake of the same cells *in vitro* using scRadiotracing. It was hypothesized that microglia display reduced relative glucose uptake when comparing *in vitro* incubation with *in vivo* glucose uptake. Enrichment by MACS facilitated the high purity of microglia (86.4% ± 3.3%), astrocytes (94.2% ± 2.4%), and microglia- and astrocyte-depleted fractions (92.6% ± 1.5%, Figure 2A). Neurons were the predominant cell type in the CD11b- and ACSA2-negative fraction, as determined by CD90.2 (79.5% ± 5.0%, Figure 2B). As expected, the isolation procedure resulted in enrichment of cell bodies, whereas cell processes, axons, and synapses were lost.

**Figure 3:**
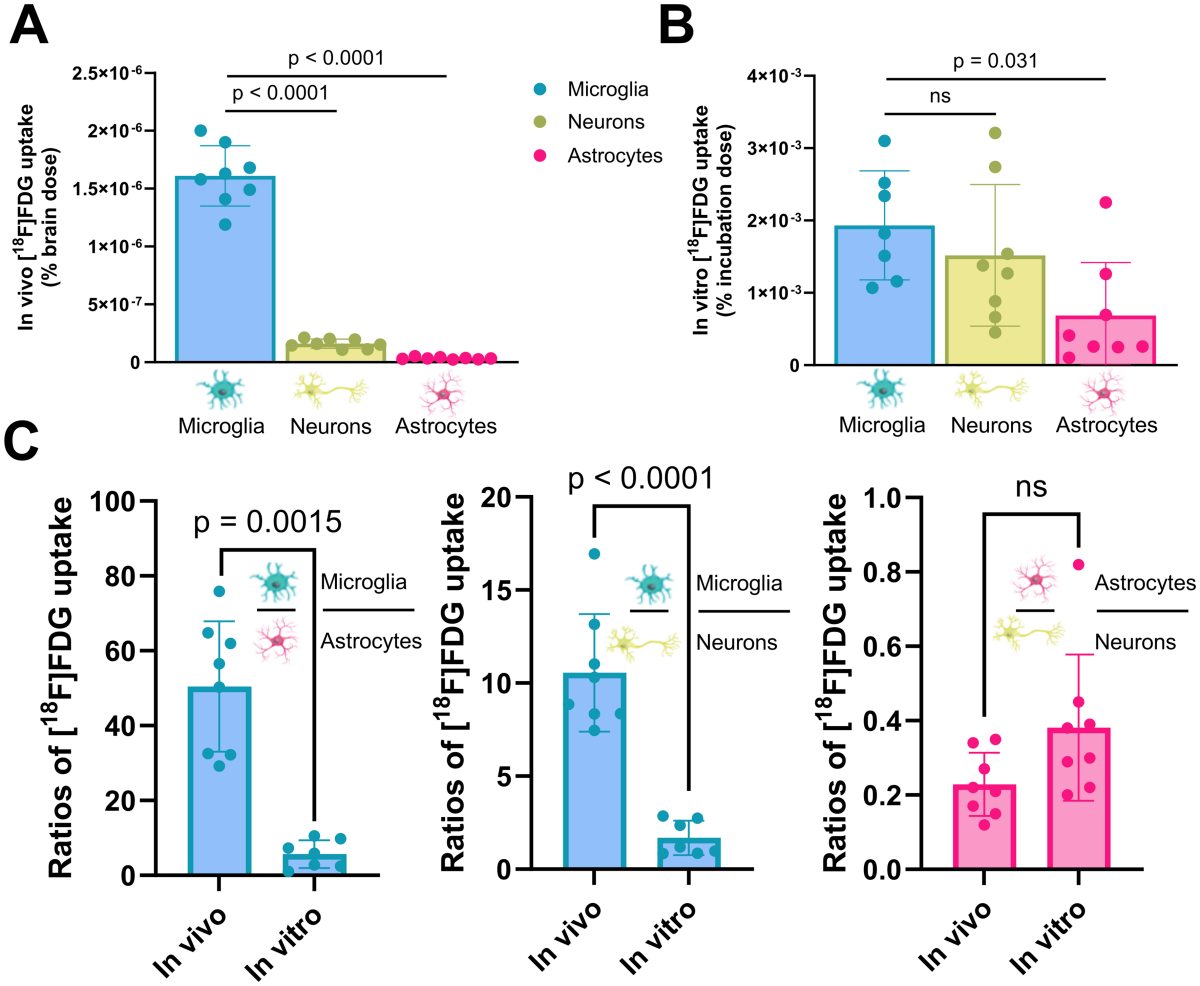
Microglia exhibit a distinct relative reduction in glucose uptake when isolated from a regular brain environment. **(A)** Direct comparison of in vivo [18F]FDG uptake among microglia, neurons, and astrocytes after normalization to whole-brain [^18^F]FDG uptake. **(B)** Direct comparison of *in vitro* [^18^F]FDG uptake among microglia, neurons, and astrocytes after normalization to the incubation dose. **(C)** Cell-type-specific ratios of [^18^F]FDG uptake in a direct comparison between *in vivo* and *in vitro* environments. After calculating the radioactivity per single cell, *in vivo* and *in vitro* microglia-to-astrocyte (left plot), microglia-to-microglia/astrocyte-negative (middle plot), and astrocyte-to-microglia/astrocyte-negative (right plot) cell ratios were compared.

After *in vivo* [^18^F]FDG injection, single microglial cell bodies showed higher glucose uptake than single astrocyte cell bodies (50.4-fold, *p* < 0.0001) and single non-microglia/non-astrocyte cell bodies (10.6-fold, *p* < 0.0001). Microglial cell bodies still showed the highest glucose uptake *in vitro*, but we observed a distinct reduction in the microglia-to-astrocyte ratio (5.7 vs. 50.4, *p* = 0.0015) and microglia-to-microglia/astrocyte-negative cell ratio (1.7 vs. 10.6, *p* < 0.0001). Glucose uptake *in vivo* was lower per astrocyte cell body than in non-microglia/non-astrocyte cell bodies (0.2-fold, p < 0.0001), and this ratio did not change significantly *in vitro* (0.4 vs. 0.2, *p* = 0.089, Figure 3).

### 3.2 Strict fasting does not affect microglial glucose uptake but increases astrocytic glucose uptake

To consider the influence of strict fasting on cells, the experiment was repeated equivalently with a glucose-free buffer (Figure 3). The mice were fasted for >3 h before tracer injection under both conditions. Subsequently, to investigate the potential effects of *in vivo* [^18^F]FDG injection, the experiment was validated using *in vitro* incubation without prior *in vivo* [^18^F]FDG injection. It was presumed that microglial glucose uptake was not influenced by fasting or prior *in vivo* [^18^F]FDG injection.

**Figure 4:**
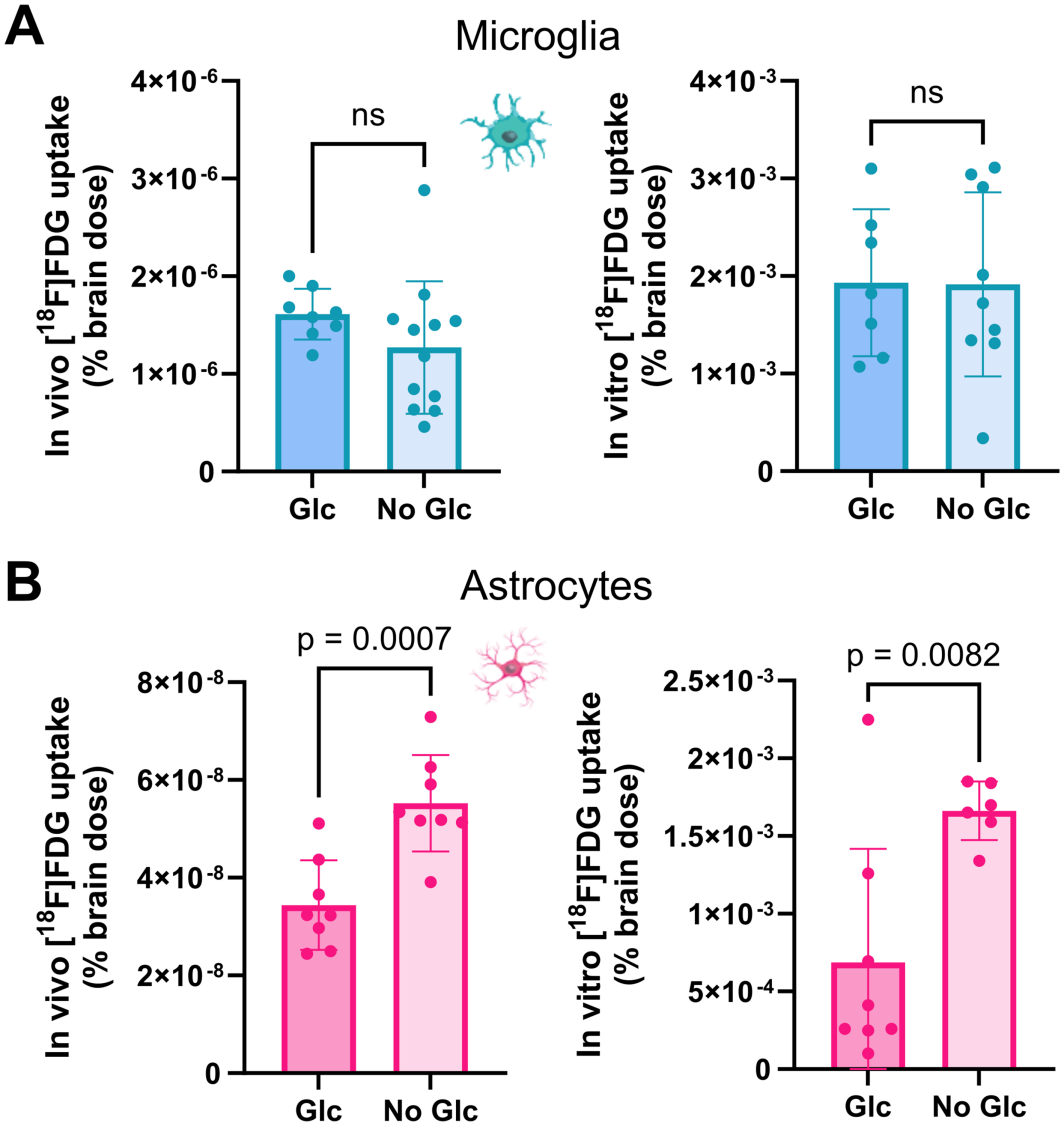
Strict fasting increases astrocytic glucose uptake but has no influence on microglial glucose uptake. Comparison of microglial and astrocytic [18F]FDG uptake in a buffer containing glucose and a glucose-free buffer. Fasting had no influence on **(A)** microglial [^18^F]FDG uptake *in vivo* (top left) or *in vitro* (top right), whereas **(B)** astrocytic [^18^F]FDG uptake increased significantly *in vivo* (bottom left) or *in vitro* (bottom right).

No significant change in glucose uptake per microglial cell body was observed when comparing glucose-free buffer with buffer containing glucose *in vivo* (1.61E-6% vs. 1.33E-6%, *p* = 0.353) or *in vitro* (1.93E-3% vs. 1.83E-3%, *p* = 0.845; Figure 4). Astrocytic glucose uptake per cell body was higher in the glucose-free buffer than in the buffer containing glucose *in vivo* (3.44E-8% vs. 5.52E-8%, *p* = 0.0007) and *in vitro* (6.85E-4% vs. 1.66E-3%, *p* = 0.0082; Figure 4). Single non-microglia/non-astrocyte cell bodies showed no significant change in glucose uptake compared with glucose-free buffer with buffer containing glucose (*in vivo:* 1.61E-7% vs. 1.43E-7%, *p* = 0.369; *in vitro*: 1.52E-3% vs. 3.48E-3%, *p* = 0.081). As a consequence, glucose-free buffer led to a reduction in the microglia-to-astrocyte ratio after *in vivo* injection (ratio 50.4 vs. 24.2, *p* < 0.0037) and *in vitro* (ratio 5.7 vs. 0.9, *p* = 0.035) compared to the buffer containing glucose. In contrast, the microglia-to-microglia/astrocyte-negative cell ratio (*in vivo:* ratio 10.6 vs. 9.1, *p* = 0.449; *in vitr*o: ratio 1.7 vs. 0.8, *p* = 0.057) remained similar, whereas the astrocyte-to-microglia/astrocyte-negative (*in vivo:* ratio 0.2 vs. 0.4, *p* < 0.0026; *in vitr*o: ratio 0.4 vs. 1.0, *p* = 0.0028) increased significantly.

Single microglia (−9%, 1.67E-3% vs. 1.83E-3%, *p* = 0.784) did not show significant changes in glucose uptake after *in vitro* incubation when compared to *in vitro* incubation after *in vivo* injection. In contrast, single astrocytes (+66%, 2.53E-3% vs. 1.66E-3%, *p* = 0.0003) indicated an increase in [^18^F]FDG uptake.

In summary, cross-validation experiments confirmed a reduction in microglial glucose uptake *in vitro* compared to *in vivo* applications. Astrocyte glucose uptake may have a higher sensitivity to low amounts of glucose than glucose uptake by microglia.

### 3.3 Relative glucose uptake of neurons, astrocytes, and microglia in cell culture differs from relative glucose uptake in the regular brain environment

**Figure 5:**
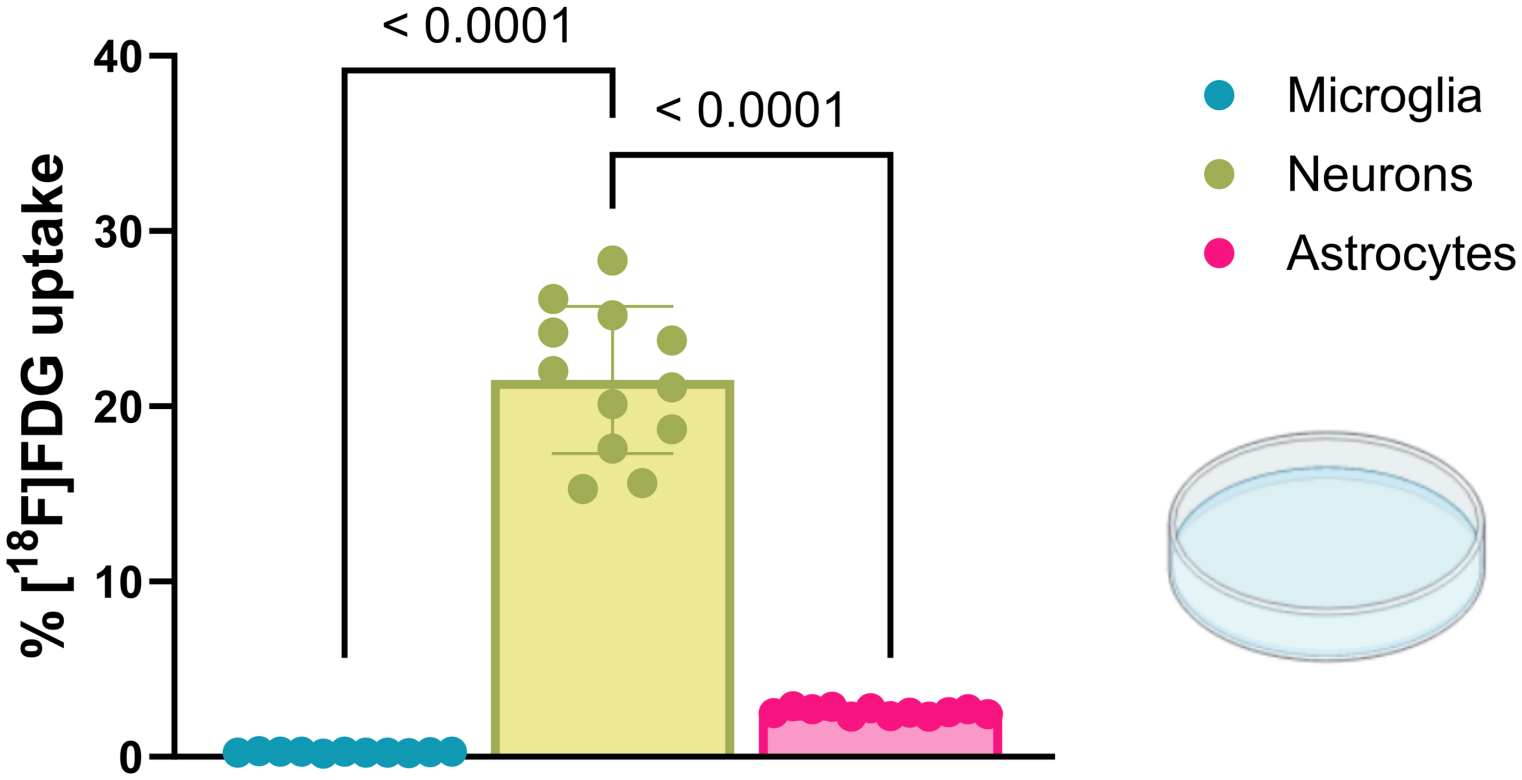
Cultured microglia, neurons, and astrocytes display altered glucose uptake compared with cells from a regular brain environment. [^18^F]FDG *in vitro* incubation showed the highest glucose uptake in cultured neurons, significantly exceeding values observed in cultured microglia and astrocytes.

Finally, we performed a cell culture experiment to compare the relative [^18^F]FDG uptake of cells isolated from the normal brain environment with that of cells in a pure *in vitro* environment (Figure 5). The rationale of this experiment was to compare the *in vitro* conditions shortly after brain dissociation (sections 3.1 and 3.2) with a pure *in vitro* environment. Neurons of the cell culture indicated the highest [^18^F]FDG uptake (10.44 ± 2.04 CPM/cell; cell fraction uptake: 21.50 ± 4.19%), exceeding astrocytes (1.25 ± 0.10 CPM/cell; cell fraction uptake: 2.57 ± 0.22%) by 8-fold (*p* < 0.0001) and microglia (0.13 ± 0.02 CPM/cell; cell fraction uptake: 0.26 ± 0.05%) by 82-fold (*p* < 0.0001).

## 4 Discussion

To study microglial glucose uptake relative to other cells of the brain in the comparison of a regular brain environment with an *in vitro* environment, we measured cellular glucose uptake in wild-type mice after *in vivo* [^18^F]FDG injection and subsequent *in vitro* incubation by scRadiotracing (Bartos et al., 2022). This novel approach provides a unique opportunity for direct head-to-head comparisons of glucose uptake in the same cells *in vivo* and *in vitro*.

Single microglial cell bodies exhibited higher glucose uptake after *in vivo* injection when compared to cell bodies of single astrocytes and cell bodies of single non-microglia/non-astrocyte cells, i.e., neurons, which was one of the main results of our previous study (Xiang et al., 2021). Despite still showing the highest glucose uptake after excessive *in vitro* [^18^F]FDG incubation, the microglia-to-astrocyte and microglia-to-microglia/astrocyte-negative cell ratios decreased distinctly in this artificial environment compared to glucose uptake in living organisms. In contrast, we did not observe any significant change in the glucose uptake ratios between astrocytes and non-microglia/non-astrocyte cells after *in vivo* injection or subsequent *in vitro* incubation. These results indicate that microglia, which are known to be highly motile in the brain (Davalos et al., 2005; Nimmerjahn et al., 2005), have reduced energy demand when they do not act in their regular brain environment. This was likely due to the lack of diverse extracellular signals. Chemoattractant triggers such as ADP or ATP (Ohsawa and Kohsaka, 2011; Davalos et al., 2005) or ligands for microglial triggering receptors, such as apolipoproteins for TREM2 (Yeh et al., 2016) regarding pathological activation, are capable of altering microglial activation states (Stence et al., 2001; Krasemann et al., 2017; Parhizkar et al., 2019). This increases synthesis and transport of neurotransmitters, requiring intermediates in the glycolytic pathway as precursor molecules (Wolosker and Mori, 2012; Dienel, 2019) and induces morphological changes in microglia, resulting in increased cellular motion (Stence et al., 2001), which also consumes glucose for ATP synthesis. In line with our previous study, the elevated [^18^F]FDG-PET signals observed in mouse models of amyloidosis were mainly driven by activated microglia (Xiang et al., 2021). Hence, supported by our current observations, *in vitro* glucose uptake values should be interpreted with caution because of the reduced cellular energy demand. Furthermore, insulin and insulin-like growth factors (IGF) are recognized as potent modulators of cellular glucose uptake. Indeed, emerging evidence indicates that insulin signaling via IGF substantially regulates microglial metabolism. In this regard, it has been demonstrated that hyperinsulinemia can alter microglial energy metabolism by impairing the membranous expression of GLUT4 (Yang et al., 2022). In addition, brain glucose metabolism was shown to be modulated by insulin, which exerts its effects on astrocytes by interacting with IGF-I (Fernandez et al., 2017). In conjunction with variations in oxygen availability, a lack of these factors *in vitro* may substantially contribute to the observed discrepancies in glucose uptake in the present study when compared to *in vivo*.

Accordingly, our results demonstrated strong differences in the relative [^18^F]FDG uptake of cultured neurons, astrocytes, and microglia *in vitro* compared to the relative glucose uptake of these cell types in the regular brain environment. Contrary to our *in vivo* results, cultured neurons exhibited by far the highest [^18^F]FDG uptake (Camandola and Mattson, 2017; Harris et al., 2012), whereas microglia showed the lowest uptake. Although the greater loss of neuronal axons and astrocytic processes during brain dissociation may partially explain this finding, the very low uptake of glucose in microglia relative to that in neurons and astrocytes should still raise awareness to interpret such results with caution. In this regard, recent data from our lab showed a strong impact of astroglial glucose uptake on changes in FDG-PET signals during aging of mice, which cannot be explained by the 82-fold and 8-fold lower glucose uptake by microglia and astrocytes compared to neurons (Bartos et al., 2024a).

In the current study, scRadiotracing was used to provide a snapshot of substrate uptake for energy metabolism (i.e., glucose) at cellular resolution, but the advantages and limitations of this methodology need to be considered. Since the results of this method are expressed as “radioactivity per cell” with normalization to the whole-brain FDG uptake, the loss of cells during the sorting procedure does not critically impact the relative analysis endpoint, regardless of whether they are unequally distributed across cell types. As an internal validation of the head-to-head comparison (*in vivo* vs. *in vitro*), we used an additional setup without *in vivo* injection to demonstrate that *in vivo* [^18^F]FDG injection prior to *in vitro* incubation did not affect microglial glucose uptake. Moreover, a possible effect of glucose-6-phosphatase activity in brain cells (Broadwell and Cataldo, 1983; Chen et al., 2017; Cossu et al., 2019; Müller et al., 2018), which hydrolyzes FDG-6-phosphate (Welch et al., 2013) and could lead to tracer washout, was reduced to a minimum by the immediate use of a cold buffer due to the enzyme temperature optimum of 37.5 °C (Ababei and Moisiu, 1970; Arcus et al., 2016).

Furthermore, we investigated the influence of strict fasting on cells during scRadiotracing, which may occur after brain dissection and thus arrests hematogenous cellular glucose supply during radioactivity measurements and brain dissociation until the media reassures a comprehensive glucose supply in the single-cell suspension. In this regard, the experiment was repeated equivalently with a glucose-free buffer, whereas the animals were fastened similarly in both setups before the tracer injection. In doing so, microglia and non-microglia/non-astrocyte cells did not show any changes in glucose uptake, either after *in vivo* injection or subsequent *in vitro* incubation. However, strict fasting appeared to affect astrocytes at least slightly by increasing their cellular glucose uptake after *in vivo* injection and *in vitro* incubation, thus suggesting a higher sensitivity to low amounts of glucose. Nevertheless, microglia still showed the highest glucose uptake *in vivo* and *in vitro* in the fasting experiment. This finding may be related to the closely linked metabolism of astrocytes and neurons. Because of the upregulation of glucose uptake and glycolysis, enabled by the protection of toxic byproducts through their glyoxalase system, astrocytes are able to compensate for their ATP demand. Simultaneously, this allows them to support neurons via the astrocyte-neuronal lactate shuttle (ANLS), which substitutes for the neuronal tricarboxylic acid cycle to maintain cellular functions and metabolism (Bélanger et al., 2011; Cleland et al., 2021; Patel et al., 2014). This assumption could be supported by obtaining no significant difference in neuronal [^18^F]FDG uptake, since neurons have no glyoxalase system compared to astrocytes (Almeida et al., 2004; Bélanger et al., 2011; Herrero-Mendez et al., 2009). However, further studies are required to elucidate the mechanisms underlying this finding.

We also considered the possible cellular saturation effects caused by prior *in vivo* [^18^F]FDG injection when performing excessive *in vitro* incubation. Our *in vitro* experiment without prior *in vivo* [^18^F]FDG injection indicated no significant changes in microglia but an increase in astrocytic glucose uptake. However, according to the literature, there is no direct evidence of negative feedback mechanisms that may arise from the phosphorylated product of [^18^F]FDG. Furthermore, because only astrocytes exhibited changes in *in vitro* glucose uptake values that were associated with varying glucose levels in the buffer used during the workflow, saturation effects caused by [^18^F]FDG-6-phosphate remain rather unlikely at the concentrations used.

Our approach to cell sorting and glucose uptake measures after *in vivo* tracer injection needs to be interpreted together with established methods of cell metabolism assessment. To understand cellular physiology, several *in vitro* methods, such as the Seahorse assay, allow real-time measurement of cellular metabolism (Caines et al., 2022; Desousa et al., 2023). The Seahorse assay quantifies cellular energy consumption using the oxygen consumption rate (OCR), which is a readout of mitochondrial respiration. Additionally, glycolysis can be monitored by measuring the extracellular acidification rate (ECAR), which reflects acidification of the medium upon lactate secretion (Divakaruni et al., 2014). However, the Seahorse assay acts in a similar *in vitro* environment as our *in vitro* FDG uptake assay. Thus, our head-to-head comparison against *in vivo* glucose uptake indicates that Seahorse results on microglia may be biased by a different metabolic state compared to the experimental conditions in living organisms.

As another method, *ex vivo* metabolomics using mass spectrometry is increasingly applied (Jang et al., 2018) to characterize energy metabolism by measuring the abundance of specific metabolites. However, to use this readout at cell-type resolution, changes in metabolites during the sorting procedure must be considered, which also accounts for the assessment of glucose transporters and enzymes by transcriptomics (Saliba et al., 2014). Interestingly, it was recently shown that cold 2-DG can be detected by mass spectroscopy (Volland et al., 2022), providing the same snapshot of the trapped glycolysis substrate as in our procedure. The advantage of mass spectroscopy is that it is available at centers without a radioprotective area, whereas scRadiotracing can provide the readout on the day of the experiment.

An important limitation of our approach using scRadiotracing is the loss of axons and processes during brain homogenization. Hence, we assume that by the measurement of cellular bodies solely, glucose uptake values in neurons and astrocytes may be underestimated. In line with this, a hypothetical model showed that only ~30% of FDG uptake in the brains of wild-type mice can be explained by known factors and cellular uptake (Xiang et al., 2022). This finding is especially important for neuronal glucose uptake, since synapses are known as the primary compartment of [^18^F]FDG uptake (Sokoloff et al., 1977; Sokoloff, 1993, 1999) and may also contribute to significantly different results when comparing scRadiotracing and cell culture data in this study. In line with this issue, we acknowledge that differences in the experimental setups between scRadiotracing and cell culture incubation (i.e., temperature difference, incubation time, and age of cultured cells) may have further amplified the observed changes. However, the potential bias is expected to be of a minor magnitude because these factors are equal to all cell fractions. Furthermore, the calculation of relative cellular uptake ratios likely balanced the impact of setup differences. Our previous study verified the dose dependency of cellular FDG uptake in neurons (Xiang et al., 2021). Hence, it was not possible to directly compare the absolute cellular glucose uptake values after *in vivo* injection with uptake values after excessive *in vitro* [^18^F]FDG incubation. Therefore, we compared the cellular uptake ratios. [^18^F]FDG uptake is also influenced by altered cellular activation states, as mentioned above. In this regard, the cell sorting procedure and subsequent cell stress may account for microglial activation, most likely resulting in increased glucose uptake during the *in vitro* incubation. In this regard, *in vitro* incubation at different time points after the sorting procedure should be tested in future investigations to study the impact of cell stress levels on glucose uptake. In addition, cellular-bound [^18^F]FDG may be released from damaged cells during brain homogenization. However, the entire workflow of scRadiotracing includes multiple dilution and washing steps, which minimize non-cellular bound tracers. Nevertheless, this could contribute to the perceived influence of fasting on astrocytic glucose uptake when a glucose-free buffer is used. Moreover, the microglia/astrocyte-negative cell fraction represents a neuron-enriched fraction with ~80% purity for comparison with the enriched microglia and astrocyte fractions. This may not precisely reflect the observed ratios in contrast to purely isolated neurons. In addition, given that the cellular marker we utilized for the validation of the neuronal proportion is also expressed on T-lymphocytes, it is evident that CD90.2 may not precisely reflect the neuronal proportion. However, since only wild-type mice were included in this study, the supposed impact of very limited amounts of T-lymphocytes on the CD90.2-positive fraction is likely negligible. Additionally, as addressed in a previous study (Bartos et al., 2024a), sex-related disparities in cellular glucose uptake need to be considered. In this regard, one study demonstrated significant alterations in cerebral GLUT expression during the estrus cycle in rats, which may affect the *in vivo* and *in vitro* uptake of glucose depending on the estrus stage (Harrell et al., 2014). Consequently, the restriction of our data to female mice limits the interpretability of the potential sex differences in microglial glucose uptake. In this regard, estrus-related changes in glucose uptake can be pronounced in living organisms, supporting the importance of *in vivo* versus *in vitro* considerations. Ultimately, it needs to be considered that isoflurane used for anesthesia is known to cause dose-dependent hypotension (Gargiulo et al., 2012), and uptake in brain cells is dependent on passing the blood–brain barrier and thus on physiological blood circulation. In this regard, isoflurane could affect relative [^18^F]FDG uptake by different cell types to a variable extent. Further studies, therefore, need to investigate additional determinants of scRadiotracing to fully understand confounding impacts on cellular glucose uptake.

In conclusion, this study demonstrated that *in vitro* measurements of microglial energy metabolism might be misleading because of the lack of influence of the *in vivo* environment. Astrocytes appear to have a higher sensitivity to low glucose levels than microglia do. For the investigation of cellular energy metabolism, there are several methods providing *in vivo* and *ex vivo* applicability. scRadiotracing facilitates substantial assessment of *in vivo* glucose metabolism, allows simple combination with other methods for extended workflows, and provides insufficient cellular resolution.

## Data Availability

The original contributions presented in the study are included in the article/supplementary material, further inquiries can be directed to the corresponding author/s.
